# The Meaning-Enhancing Properties of Psychedelics and Their Mediator Role in Psychedelic Therapy, Spirituality, and Creativity

**DOI:** 10.3389/fnins.2018.00129

**Published:** 2018-03-06

**Authors:** Ido Hartogsohn

**Affiliations:** Science, Technology and Society Program, Harvard Kennedy School, Harvard University, Cambridge, MA, United States

**Keywords:** psychedelics, hallucinogens, mediators, meaning, placebo, therapy, mystical-experience, creativity

## Abstract

Past research has demonstrated to the ability of psychedelics to enhance suggestibility, and pointed to their ability to amplify perception of meaning. This paper examines the existing evidence for the meaning-enhancing properties of psychedelics, and argues that the tendency of these agents to enhance the perception of significance offers valuable clues to explaining their reported ability to stimulate a variety of therapeutic processes, enhance creativity, and instigate mystical-type experiences. Building upon previous research, which suggested the potential role of psychedelic meaning-enhancement in enhancing placebo response, the paper explores the mechanisms by which the meaning-amplifying properties of psychedelics might also play a role in enhancing creativity, as well as in effecting mystical-type experiences. The wider social and public-health implications of this hypothesis are discussed, and suggestions are made as to the various ways in which scientific understanding of the meaning-enhancing properties of psychedelics might be advanced and utilized.

How do psychedelics induce their dramatic and variegated effects which include, among other phenomena, psychotherapeutic insights, creative breakthroughs, and mystical-type experiences? The recent literature on psychedelics showcases a growing preoccupation with the underlying mechanisms responsible for the panoply of extraordinary effects instigated by these agents. Some recent papers have sought to offer a grand unifying theory of psychedelic action by pointing to neuropharmacological mechanisms that underlie psychedelic action (Carhart-Harris and Nutt, 2017; Nichols et al., 2017). Others have focused their attention on the role of psychological mediating factors in determining reactions to psychedelics. The most commonly mentioned of these mediators of psychedelic response are unquestionably spiritually meaningful experiences and experiences of ego dissolution, whose occurrence is often correlated with the success of therapy, but researchers have also suggested a numbers of other mediators including relational embeddness, embodiment, “the difficult struggle,” affect and catharsis, visions, and recovered sense of appropriate priorities (Griffiths et al., 2006; Lebedev et al., 2016; Ross et al., 2016; Belser et al., 2017; Johnson et al., 2017). In this paper I wish to argue for the importance of another often overlooked mediator of psychedelic action, which is fundamental to understanding the effects of psychedelics in therapy, creativity, and spirituality. I am referring to the remarkable tendency of these agents to enhance the perception of meaning, or, in other words, to cause things to appear dramatically more meaningful than they would otherwise seem to be.

Extraordinarily, though the ability of psychedelics to enhance perception of meaning is supported by the literature, it has so far not been the focus of any deliberate and sustained line of inquiry (For current research supporting the meaning-enhancing properties of psychedelics see Carhart-Harris et al., 2014; Kaelen et al., 2015; Preller et al., 2017). Nevertheless, hints and traces of this idea and its significant implications permeate both popular and clinical psychedelic literature. In Huxley's *Doors of Perception*, the classic text that brought psychedelics to popular attention in the West, the author noted that under the effects of the drugs objects were “all but quivering under the pressure of significance by which they were charged,” (Huxley, 2011, p. 17–18; For a similar observation see Cohen, 1964, p. 90) and made enlightening observations about psychedelic aesthetics and its crucial relation to radical alterations in the perception of meaning (Huxley, 2011, p. 95; for similar observations see also Belser et al., 2017, p. 379). Clinical research has also provided data to support the claim that psychedelics enhance the perception of meaning (Carhart-Harris et al., 2014; Kaelen et al., 2015; Preller et al., 2017). Remarkably, one of the most striking qualities of psychedelics, which was noted on by both 1960s as well as by contemporary psychedelic researchers, is their ability to induce experiences which people regard as extremely meaningful. Between two thirds to 86% of those who have psychedelic experiences in a supportive therapeutic setting consider them to be either one of the five most meaningful and spiritually significant experiences of their lives, or the single most meaningful experience (Griffiths et al., 2006; Leary, 2007; Johnson et al., 2017). Proving the clinical efficacy of psychedelics has posed considerable challenges for psychedelic researchers for decades, yet the ability of these agents to reliably elicit subjectively-perceived highly meaningful experiences is beyond doubt.

While this in itself constitutes no proof, it is telling that the very word “psychedelic” (from the Greek Psykhe-psyche + Deloun-reveal, manifest) seems to quite straightforwardly convey the idea that psychedelics enhance the perception of meaning. The widely accepted designation of these substances as “mind-manifesting” or “mind-revealing” speaks to their ability to enhance and accentuate (by “manifesting” or “revealing”) whatever objects exist in the mind^1^. Psychedelics have commonly been described as *magnifiers, amplifiers*, and *augmenters* of consciousness (Schneider, 1967; Lee and Shlain, 1992, p. 200; Higgs, 2006, p. 54; Grof, 2008; Strassman et al., 2008, p. 148). What these overlapping, perhaps even coterminous, terms seem to share is the recognition that psychedelics intensify mental phenomena and cause them and their significance to appear bigger, vaster, and more dramatic than otherwise.

Additional evidence of the widespread recognition that a key aspect of psychedelic efficacy relates to the agents' ability to enhance the perception of meaning can be found in the growing discourse contrasting the dissimilar modes in which psychedelics and SSRI-anti depressants treat depression (Carhart-Harris and Goodwin, 2017; Roseman, 2017; Watts et al., 2017). Several recent papers present SSRI medication as agents that diminish the intensity of experience, thereby allowing individuals who are otherwise overwhelmed by feelings to adequately cope and function. In these and similar accounts, SSRI's are regarded as commensurate with a less dramatic, more flattened experience of the world. Psychedelics, by contrast, are regularly described in these and other accounts as doing the exact opposite: as drugs which amplify consciousness, and augment the intensity of perception, emotional reactions, and neurological indicators such as amygdala response. Without engaging the validity of such ideas, this discourse, which brings psychedelics in conjunction with another, allegedly diametrically opposed family of psychoactives, provides further evidence of the widely held view that psychedelics enhance perception of meaning. SSRI therapy, it argues, functions by diminishing emotional volume, thereby making experiences more bearable, while psychedelic therapy functions by amplifying emotional volume and demanding that patients “face the demon.”

Examining psychedelics through the prism of their ability to enhance perception of meaning provides valuable insights into their remarkable effects by allowing a keener appreciation of the different ways in which amplification of experience shapes key aspects and characteristics of psychedelic action. More specifically, the meaning-enhancing effects of psychedelics seems to play a key role in the fields of therapy, spirituality, and creativity enhancement.

Coming first to the issue of therapy. In a recent paper I have argued that a substantial part of the therapeutic effects of psychedelics might be explained by bringing psychedelic theory into contact with the growing field of placebo research (Hartogsohn, 2016). This relation becomes evident when one considers the fact that placebo researchers have proposed the concept of “meaning response” as a more accurate term to replace the arguably problematic term “placebo” (Moerman, 2002). The concept of “meaning response” advances the idea that subjective experiences of knowledge, symbol, and meaning can have pronounced biological, and medically therapeutic effects of the type commonly described as “placebo” (Moerman, 2002, p. 4). The amplification of meaning by psychedelics therefore automatically entails amplification of placebo, and can offer help in explaining psychedelics' extraordinarily versatile uses and applicability in a wide variety of medical conditions (Hartogsohn, 2016; Strassman, 2017). Understanding psychedelics as enhancers of meaning-response also explains why the concept of *Set and Setting*—a doctrine for the beneficial management of meaning response—has emerged within psychedelic research (Hartogsohn, 2017). The psychological context (set) and sociocultural context (setting) of a psychedelic experience are considered crucial because their meaning is significantly multiplied by the effects of the drugs, rendering each and any factor of extreme importance. The relevance of set and setting, and the validity of the psychedelics-as-placebo-enhancers approach can be clinically tested by controlling for variables such as expectation, intention, and doctor-patient relationship (for more concrete on how this might be done see Hartogsohn, 2016, 2017; Strassman, 2017).

Mystical-type experiences are also enhanced by the meaning-enhancing properties of psychedelics. Evidence of the ability of psychedelics to induce spiritually significant experiences were provided in Pahnke's 1962 Good Friday Experiment, whose results were later corroborated by recent studies into the mysticomimetic qualities of psychedelics (Pahnke, 1963; Doblin, 1991; Griffiths et al., 2006; Bossis, 2017). Crucially, one of the four principal features of such mystical-type experiences, as defined by James, is their noetic quality, i.e., the experience of gaining access to a profounder, more significant plane of existence imbued with paramount authority and significance which transcend ordinary reality (James, 1985). It is highly conceivable that such noetic qualities would be strengthened by meaning-enhancement. Similarly, conversion experiences are often triggered by a sense of encounter with a formidable, awesome, “greater-than-human” presence that radiates immense significance and meaning—an encounter with an hyperreal dimension of overblown metaphysical proportions, which some religious scholars classically referred to as the *numinous* or *mysterium tremendum* (Otto, 1958; Merkur, 2006). This experience of confronting an overwhelming, ineffable, and even unfathomable quality of the world is arguably facilitated by the tendency of psychedelics to imbue the mind and the external world with vibrant significance, as noted by Huxley (2011, p. 17–18). By causing mental and external phenomena to appear immensely more significant, psychedelics facilitate magical thinking and a reenchanted experience of the world (Carhart-Harris, 2013). Crucially, the perception of the significance of psychedelically induced mystical-type experiences is magnified as well. In other words, mystical-type experiences and insights obtained on psychedelics subjectively appear more significant than comparable non-psychedelically induced experiences and insights by virtue of the meaning-enhancing action of these drugs.

Meaning-enhancement arguably plays an additional role in psychedelically induced enhancement of creativity and problem-solving capabilities. While research on psychedelic enhancement of creativity is scant, largely dated, and often inconclusive (Baggott, 2015) some evidence does point to the creativity enhancing properties of psychedelics. Beyond popular lore which credits psychedelics for the performance of great creative feats (Bromell, 2002; Markoff, 2005), several clinical studies have indicated creativity improvements following psychedelic use, while others have called for the renewal of research into the creative benefits of psychedelic use (Harman et al., 1966; De Rios et al., 2003; Sessa, 2008; Kuypers et al., 2016). Here as well, meaning enhancement might play a key role. By magnifying the perceived significance of creative challenges and insights psychedelics provide users with the impetus to pursue new, less obvious lines of ideation that they might otherwise have ignored; and with enhanced motivation to explore new creative directions to their fullest ramifications. Some investigators have noted the potential role of meaning finding in enhancing creativity (Barron, 1965) while others have pointed to the importance of other mediators that could arguably be correlated with enhanced perception of meaning such as reduced inhibition or heightened empathy (Harman et al., 1966). By imbuing possible solutions with a magnified sense of meaning and plausibility, psychedelics might assist in reducing inhibitions, self-criticism, and kindle greater concentration and enthusiasm for creative exploration. Crucially, here as well, psychedelics might also enhance the perceived significance of such creative breakthroughs. As with spiritual experiences, this is not to say that creative-breakthroughs with psychedelics are invalid, but that one should be aware of the tendency to overstate the importance of such breakthroughs, particularly during, or shortly after psychedelic experience.

Finally, it should be noted that the meaning-intensifying properties of psychedelics also play a key role in precipitating what has been described as their psychotomimetic or psychosis inducing properties. The increased intensity that psychedelics bring to experience, and the increased significance with which they imbue mental objects can manifest itself equally in spiritual epiphanies as well as in paranoid thought patterns, intensified anxieties, amplified fantasies, and other pathological thought patterns (Carhart-Harris, 2013).

## Discussion

How does the recognition of the meaning-enhancing role of psychedelics alter our perception of these agents and their utility? One implication is to allow us a clearer understanding of their mode of action, of the potential outcomes of psychedelic experiences, as well as of the ways in which deliberate use of such meaning-enhancing qualities might assist therapy, enhance religious life, and facilitate creative activity.

From a wider theoretical perspective, psychedelics' function as enhancers of meaning can be seen in the broader cultural context of late modernity's struggle to make sense and meaning of life in increasingly atomized, individualized, and stress-ridden societies; a difficulty compounded by the disappearing role of religion and the implosion of linear narratives of progress (Bauman, 2001; Putnam, 2001; Alexander, 2010). Philosophers and sociologists have long warned that life in industrialized, technological societies is undergoing a process of impoverishment of meaning (Ellul, 1967; Marcuse, 1991; Alexander, 2010). Such tendencies might be brought in conjunction with growing empirical data on the rising prevalence of depression, suicidality, and other psychopathologies in modern societies (Hagnell et al., 1994; Healy, 2002, p. 371–372; Andrade et al., 2003; Twenge et al., 2010; Hidaka, 2012; PMC, 2013; Curtin et al., 2016). Several studies have demonstrated correlations between feelings of meaning in life and increased psychological well-being, increased longevity, as well as reduced risk of suicidality and depression (Boyle et al., 2009; Ho et al., 2010; Park et al., 2010; Carlo et al., 2011; McMahan and Renken, 2011; Kleiman and Beaver, 2013). Perhaps, says Michael Steger, who studies the psychology of meaning, “meaning is a matter of life and death” (Steger, 2013). Could psychedelics help fight rising rates of psychopathologies by bolstering individual and social sense of meaning and purpose? We are unquestionably still far from answering such questions, but evidence does point to potentially significant implications which the psychedelic meaning-enhancement model might have for fortifying society's resistance to mental pathology.

Finally, considering the utility of psychedelics for the enhancement of sense meaning, certain metaphysical questions might enter the discussion. Namely, is it ethically acceptable to artificially bolster the meaning of experiences and relationships? Some might argue that the ability of psychedelics to amplify meaning beyond its normal dimensions turns them into nothing else than mental illusogens that create only illusions of profoundness. When drugs cause their users to find more meaning in their experiences and relationships than ordinary circumstances allow, might this represent an insidious form of self-deception?

The argument seems compelling at first, yet it is arguably flawed. It relies on the assumption that there exists one “correct” mental framework from which to approach the world and that any psychochemically induced digression from that norm is inherently wrong. In practice, human ability to meaningfully relate and to authentically appreciate experiences is contingent on myriad factors of everyday life, and arguably strongly disrupted by the circumstances of life within atomized, competitive, high-stress, bureaucratized societies. The psychedelic perspective could thus be viewed along a two-poled spectrum which runs the gamut from utter depletion of meaning to overwhelming abundance of meaning.

The question, then, is whether there is anything inherently wrong in using a chemical to find more meaning in one's life or in a close human relationship even when such artificially-stimulated insights of newly found intimacy and authenticity continue to prove themselves meaningful and helpful in the long run, as demonstrated by studies? (Griffiths et al., 2008; Lebedev et al., 2016; Ross et al., 2016; Johnson et al., 2017). This is an intriguing and arguably normative issue which should not be left for medicine to decide, and it is rendered moot in cases where pathology and deep suffering is involved, as can be seen by the ample use of psychotropic medicine in contemporary psychiatry.

## Conclusion

The meaning-enhancement property of psychedelics is a hypothesis supported by classic accounts of the psychedelic experience as well as by clinical research, but it has not yet received the attention it deserves. This path of investigation can be opened up by employing various psychometric tools to help assess the degree to which psychedelics enhance meaning and potential correlations with therapeutic, spiritual, or creative benefits. Recent, initial and still unpublished results have found significant increase in meaning in life following administration of psilocybin, as measured by the Meaning in Life Questionnaire (Personal communication, Leor Roseman and Eline Haijen, Nov 22, 2017). Future research might develop and employ similar questionnaires to study the degree to which psychedelics might amplify the perceived meaning of objects, activities, emotions, thoughts, and beliefs. Demonstrating the meaning-enhancing effect of psychedelics can advance our understanding of psychedelic effects and offer new paths for investigation and use in the fields of therapy, religion, and creativity.

## Author contributions

The author confirms being the sole contributor of this work and approved it for publication.

### Conflict of interest statement

The author declares that the research was conducted in the absence of any commercial or financial relationships that could be construed as a potential conflict of interest.

## Footnotes

^1^Similarly, their designation as “ecodelics,” suggested by Richard Dolye, which signifies their ability to accentuate or enlarge the significance of external objects.
